# The MapZ-Mediated Methylation of Chemoreceptors Contributes to Pathogenicity of *Pseudomonas aeruginosa*

**DOI:** 10.3389/fmicb.2019.00067

**Published:** 2019-02-05

**Authors:** Shuo Sheng, Lingyi Xin, Joey Kuok Hoong Yam, May Margarette Salido, Nicole Zi Jia Khong, Qiong Liu, Rachel Andrea Chea, Hoi Yeung Li, Liang Yang, Zhao-Xun Liang, Linghui Xu

**Affiliations:** ^1^Guangdong Province Key Laboratory of Microbial Signals and Disease Control, Integrative Microbiology Research Centre, South China Agricultural University, Guangzhou, China; ^2^Guangdong Innovative and Entrepreneurial Research Team of Sociomicrobiology, South China Agricultural University, Guangzhou, China; ^3^School of Biological Sciences, Nanyang Technological University, Singapore, Singapore; ^4^Singapore Centre for Environmental Life Sciences Engineering, Nanyang Technological University, Singapore, Singapore; ^5^Interdisciplinary Graduate School, Nanyang Technological University, Singapore, Singapore

**Keywords:** *Pseudomonas aeruginosa*, chemotaxis, PilZ adaptor, cyclic di-GMP, methyl-accepting chemotaxis protein, methyltransferase cheR1, bacterial pathogenesis

## Abstract

The pathogenic bacterium *Pseudomonas aeruginosa* is notorious for causing acute and chronic infections in humans. The ability to infect host by *P. aeruginosa* is dependent on a complex cellular signaling network, which includes a large number of chemosensory signaling pathways that rely on the methyl-accepting chemotaxis proteins (MCPs). We previously found that the second messenger c-di-GMP-binding adaptor MapZ modulates the methylation of an amino acid-detecting MCP by directly interacting with a chemotaxis methyltransferase CheR1. The current study further expands our understanding of the role of MapZ in regulating chemosensory pathways by demonstrating that MapZ suppresses the methylation of multiple MCPs in *P. aeruginosa* PAO1. The MCPs under the control of MapZ include five MCPs (Aer, CtpH, CptM, PctA, and PctB) for detecting oxygen/energy, inorganic phosphate, malate and amino acids, and three MCPs (PA1251, PA1608, and PA2867) for detecting unknown chemoattractant or chemorepellent. Chemotaxis assays showed that overexpression of MapZ hampered the taxis of *P. aeruginosa* toward chemoattractants and scratch-wounded human cells. Mouse infection experiments demonstrated that a dysfunction in MapZ regulation had a profound negative impact on the dissemination of *P. aeruginosa* and resulted in attenuated bacterial virulence. Together, the results imply that by controlling the methylation of various MCPs via the adaptor protein MapZ, c-di-GMP exerts a profound influence on chemotactic responses and bacterial pathogenesis.

## Introduction

The opportunistic pathogen *Pseudomonas aeruginosa* is capable of causing severe and potentially lethal infections in immunocompromised patients. *P. aeruginosa* respond to a wide range of chemical attractants and repellents by transducing the chemical signal into flagellar responses to change the direction of motor rotation. The tight coupling between chemotaxis and motor output enables the bacterial cells to migrate toward the source of a chemoattractant or away from a repellent. The chemotaxis-controlled flagellar output is not only essential for the survival of *P. aeruginosa* in diverse environment, but also crucial for the bacterial infection process by promoting dissemination and biofilm formation (Sampedro et al., 2015; Matilla and Krell, 2018). In the heart of a bacterial chemotaxis system, a mosaic of methyl-accepting chemotaxis proteins (MCPs) form the membrane-embedded chemoreceptor array that binds and detects the attractants and repellants. MCPs bind the attractants or repellents and transduce the chemical information to the autokinase CheA via the scaffolding protein CheW. A decrease in attractant-binding activates the autokinase activity of CheA and an increase in repellent-binding suppresses the autokinase activity. When activated, CheA undergoes autophosphorylation and transfers the phosphoryl group to CheY. Subsequent binding of phosphorylated CheY to the flagellar rotor changes the direction of flagellar rotation, causing a reversal in swimming direction for the mono-flagellated *P.aeruginosa*. The ligand-binding activity of MCPs is modulated by a methyltransferase (CheR) and a methylesterase (CheB), with CheR methylating specific glutamyl residues in MCPs and CheB removing the methyl groups. CheR and CheB constitutes an adaptation mechanism that constantly resets the MCPs to a pre-stimulus state as the bacterium travels through a ligand gradient. This adaptation mechanism allows MCPs to monitor changes over a wide range of concentrations and to enable the bacterium to move up or down the concentration gradient.

*Pseudomonas aeruginosa* PAO1 has two chemotaxis pathways (Che I, Che II) involved in flagellum-mediated chemotactic responses (Ortega et al., 2017). While the Che I chemotaxis pathway is essential for chemotaxis, the Che II pathway seems to be required for fine-tuning chemotaxis under certain conditions (Ferrandez et al., 2002). The genome of *P. aeruginosa* PAO1 encodes four chemotaxis methyltransferases, with CheR1 as the main methyltransferase mediating flagellum-dependent chemotaxis. It was discovered recently that c-di-GMP, a second messenger found *in P. aeruginosa* and many other bacteria, inhibits the methyltransferase activity of CheR1 through the adaptor protein MapZ to suppress the methylation of the amino acid-sensing MCP PctA (Xu et al., 2016; Yan et al., 2018). The modulation of MCP methylation by c-di-GMP affects the autokinase activity of CheA, phosphorylation of the CheY-like proteins and chemotactic responses. The finding that c-di-GMP inhibits the methyltransferase activity of CheR1 implies that the chemotactic response in *P. aeruginosa* is modulated by cellular c-di-GMP concentration. Considering that the CheA autokinase activity is known to modulate cellular c-di-GMP concentration (Kulasekara et al., 2013), the findings also suggest that the chemosensory and c-di-GMP signaling systems are likely to interact with each other in a reciprocal manner.

The genome of *P. aeruginosa* PAO1 harbors 23 genes that encode membrane-bound MCP-like proteins. Among the MCPs, PA2561(CtpH)/PA4844 (CtpL) (Wu et al., 2000; Rico-Jimenez et al., 2016), PA2652(CtpM) (Alvarez-Ortega and Harwood, 2007; Martin-Mora et al., 2018), PA4309 (PctA)/PA4310 (PctB)/PA4307 (PctC) (Taguchi et al., 1997; Rico-Jimenez et al., 2013), PA1561 (Aer, also formerly known as TlpC) (Hong et al., 2004), PA0176 (Aer2) (Hong et al., 2004), PA2654(TlpQ) (Kim et al., 2007), PA0180(CttP) (Kim et al., 2006), and PA5072(McpK) (Martin-Mora et al., 2016) have been shown to be responsible for detecting ligands such as inorganic phosphate, malate, amino acids and gamma aminobutyrate (GABA), oxygen, ethylene, chloroethylenes, and α-ketoglutarate. Currently, it is not known how many of the 23 MCPs are methylated by CheR1, and thus, subjected to the control of MapZ and c-di-GMP. To further advance our understanding on the regulation of chemosensory pathways by the MapZ-mediated mechanism in *P. aeruginosa*, we identified all the MCP substrates for CheR1 using *in vitro* methylation assay. We demonstrated that MCP methylation is inhibited by MapZ in the presence of c-di-GMP. The recent finding that *P. aeruginosa* requires CheR1 and flagellum-mediated chemotaxis for fitness and virulence in acute infections prompted us to examine the role of MapZ in bacterial infection (Turner et al., 2014). We found that a dysfunction in MapZ regulation decreased the efficiency of chemotaxis and resulted in attenuated bacterial pathogenicity. Taken together, the results suggest that MapZ-mediated mechanism has a profound influence on chemotactic responses and bacterial pathogenicity by controlling the methylation and activity of multiple MCPs.

## Results

### CheR1 Catalyses the Methylation of Multiple MCPs in *P. aeruginosa* PAO1

**Figure 1:**
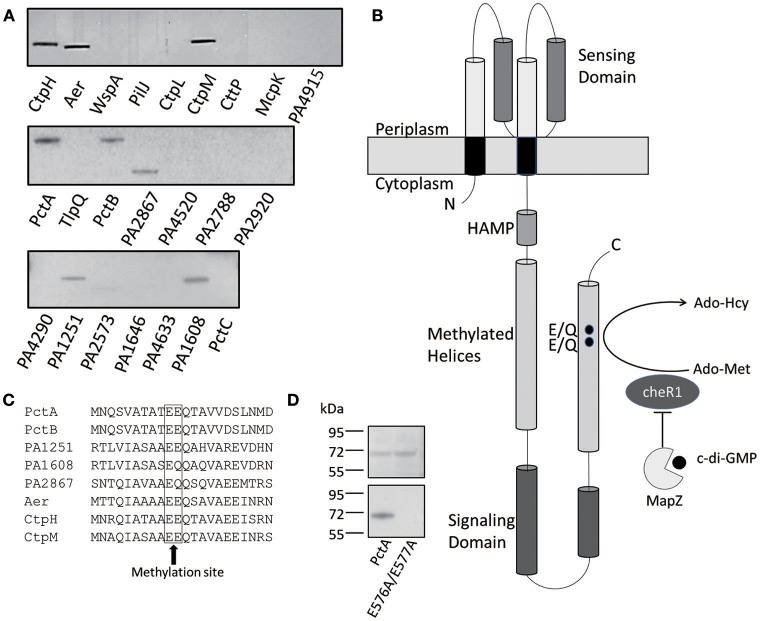
CheR1-catalyzed methylation of eight MCPs. **(A)** Methylation of eight MCPs by CheR1 demonstrated by *in vitro* methyltransferase assay with the methylated MCPs visualized by radioautography. The membrane fraction-containing chemoreceptor and [^3^H] Ado-Met were used as the substrate and co-substrates for CheR1, respectively. The amount of membrane fraction was normalized according to the corresponding protein expression level. Data are representative of two independent experiments. **(B)** Schematic representation of the architecture of PctA with the ligand-binding domain located in the periplasm and the cytoplasmic portion comprised of a HAMP domain, methylated helices and a signaling domain. The MapZ-mediated inhibition of CheR1 by c-di-GMP is depicted along with the predicted methylation sites (E/Q) in the methylated helices. **(C)** Partial sequence alignment of the eight MCP substrates of CheR1 with the predicted methylation sites are shown in the frame. **(D)** *In vitro* methyltransferase assay shows the methylation of PctA, but not PctA_E576AE577A_, by CheR1. The membrane fraction-containing MCP and [^3^H] Ado-Met were incubated with CheR1 before the methylated MCP was visualized by radioautography. SDS-PAGE gel (upper panel) shows that the amount of MCP proteins used in the assay were comparable. The methylated MCPs were visualized by radioautography (lower panel). Data are representative of two independent experiments.

It was shown previously that the chemotaxis methyltransferase CheR1 of *P. aeruginosa* methylates PctA, one of the MCPs involved in sensing amino acids (Schmidt et al., 2011). There are 22 more membrane-bound MCPs in *P. aeruginosa* PAO1 and it was not known how many of the MCPs are methylated by CheR1. We cloned PctA and other 22 MCP genes individually into the overexpression plasmid pHSe5 and expressed the MCPs in the *E. coli* HCB721 strain. Three MCPs [PA1930, PA1423(BdlA), PA0176(McpB)] without membrane-bound domain were not included in this study. The *E. coli* HCB721 strain, which had all the endogenous *mcp* genes deleted, was originally created by Berg (Wolfe et al., 1988) and co-workers to investigate the function of MCPs. Using the overexpression plasmids, all 23 MCPs were expressed and detected in the HCB721 host. We collected the membrane fraction using the method of sucrose gradient ultracentrifugation following the established procedure (Xu et al., 2016); and performed *in vitro* methylation assays by incubating the recombinant CheR1, ^3^H-labeled S-adenosyl methionine (Ado-Met) and each MCP-containing membrane fraction with the amount of protein normalized according to the protein expression level. After a 30 min incubation, radioactivity was detected for eight of the 23 MCPs that include Aer, CtpH, CtpM, PctA, PctB, PA1251 PA1608, and PA2867 (Figure 1A). Differences in the intensity of the bands (i.e., radioactivity) were observed for the eight MCPs, indicating that CheR1 possesses some substrate preference toward the MCPs. Those eight chemoreceptors methylated by methyltransferase CheR1 belong to the Che I/F6 pathway controlling flagella-mediated motility (Ortega et al., 2017). The two MCPs (PA0180 and PA4290) that do not contain the methylation sites were not methylated in our experiments as expected. The two MCPs (WspA and PilJ) which belong to evolutionary class Wsp/ACF or Chp/TCF respectively were also not methylated in our assay (Ortega et al., 2017).

Most MCPs from *P. aeruginosa* PAO1 contain one or two glutamyl residues as the putative methylation sites within the (A/S)X(A/T)(A/T/C/S)**E**(**E**/Q)Q motif found in the C-terminal region of the signal transduction domain (Figure 1B; Alexander and Zhulin, 2007). All the eight MCPs methylated by CheR1 contain the (A/S)X(A/T)(A/T/C/S)**E**(**E**/Q)Q motif (Figure 1C). To validate the two glutamyl residues are indeed the methylation sites in PctA, we cloned and expressed a PctA double mutant with residues Glu^576^ and Glu^577^ replaced with Ala using the *E. coli* HCB721 host. *In vitro* methylation assay showed the PctA^E576A/E577A^ mutant protein could not be methylated when incubated with CheR1 and ^3^H-labeled Ado-Met (Figure 1D), confirming that the two glutamyl residues are the likely methylation sites.

### MapZ Inhibits CheR1-Catalyzed Methylation of MCPs in the Presence of C-di-GMP

**Figure 2:**
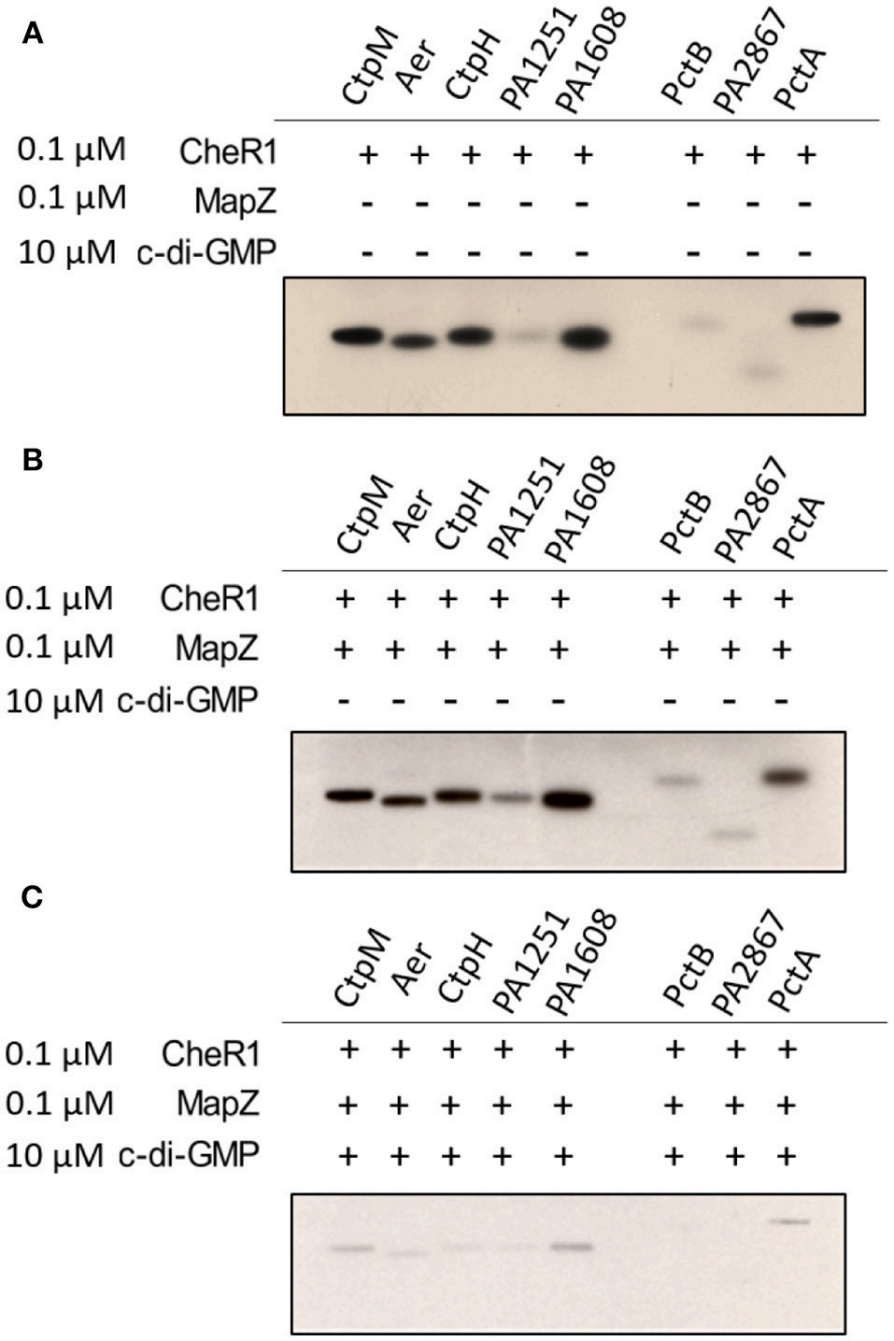
Inhibition of CheR1 by c-di-GMP in methylating MCPs requires MapZ. Methylation of eight MCPs by CheR1 demonstrated by *in vitro* methyltransferase assay with the methylated MCPs visualized by radio-autography. Shown radio-autography images **(A–C)** are representative of two independent *in vitro* methyltransferase assay. The membrane fraction-containing chemoreceptor and [^3^H]-Ado-Met were used as the substrate and co-substrates for CheR1, respectively. The amount of membrane fraction was normalized according to the corresponding protein expression level.

We recently found that MapZ inhibits CheR1 to suppress the methylation of the chemoreceptor PctA in the presence of the c-di-GMP (Xu et al., 2016). Here we performed *in vitro* methylation assays to test whether MapZ/c-di-GMP also inhibits CheR1-catalyzed methylation of the other seven MCPs. When the MCP-containing membrane fractions were incubated with CheR1 and [^3^H]-Ado-Met, methylation of the eight MCPs was observed (Figure 2A). Inclusion of MapZ in the reaction mixture did not seem to affect the methylation level of the MCPs (Figure 2B), suggesting that MapZ alone does not inhibit CheR1. In contrast, addition of MapZ and 10 μM c-di-GMP together to the reaction mixture resulted in significantly lower methylation levels, suggesting that the methylation of MCP was inhibited in the presence of MapZ and c-di-GMP (Figure 2C). The observations are consistent with our previous observation that the inhibition of CheR1 by MapZ is dependent on the presence of c-di-GMP. Together, the *in vitro* results suggest that MapZ and c-di-GMP together can inhibit the methylation of multiple MCPs.

### High Cellular Level of MapZ Negatively Affects the Efficiency of Chemotaxis and Energy-Taxis

As a part of the adaptation mechanism, temporal regulation of the methylation level of MCPs is crucial for resetting the autokinase activity of CheA to achieve efficient chemotaxis. While the ligands for three (PA1251, PA1608, and PA2867) of the eight MCPs methylated by CheR1 remain unknown, the ligands for the other five MCPs have been identified as amino acids (PctA, PctB), phosphate (CtpH), malate (CtpM), and oxygen or energy level (Aer) (Hong et al., 2004; Alvarez-Ortega and Harwood, 2007; Schweinitzer and Josenhans, 2010; Rico-Jimenez et al., 2016). The control of c-di-GMP and MapZ on the PctA/B-mediated chemotaxis toward amino acids was demonstrated previously, with the overexpression of MapZ decreasing the methylation of PctA to weaken the chemotactic response to l-serine (Xu et al., 2016). Here we examine how MapZ affects the chemotactic ability of *P. aeruginosa* in response to phosphate, malate as well as energy taxis.

**Figure 3:**
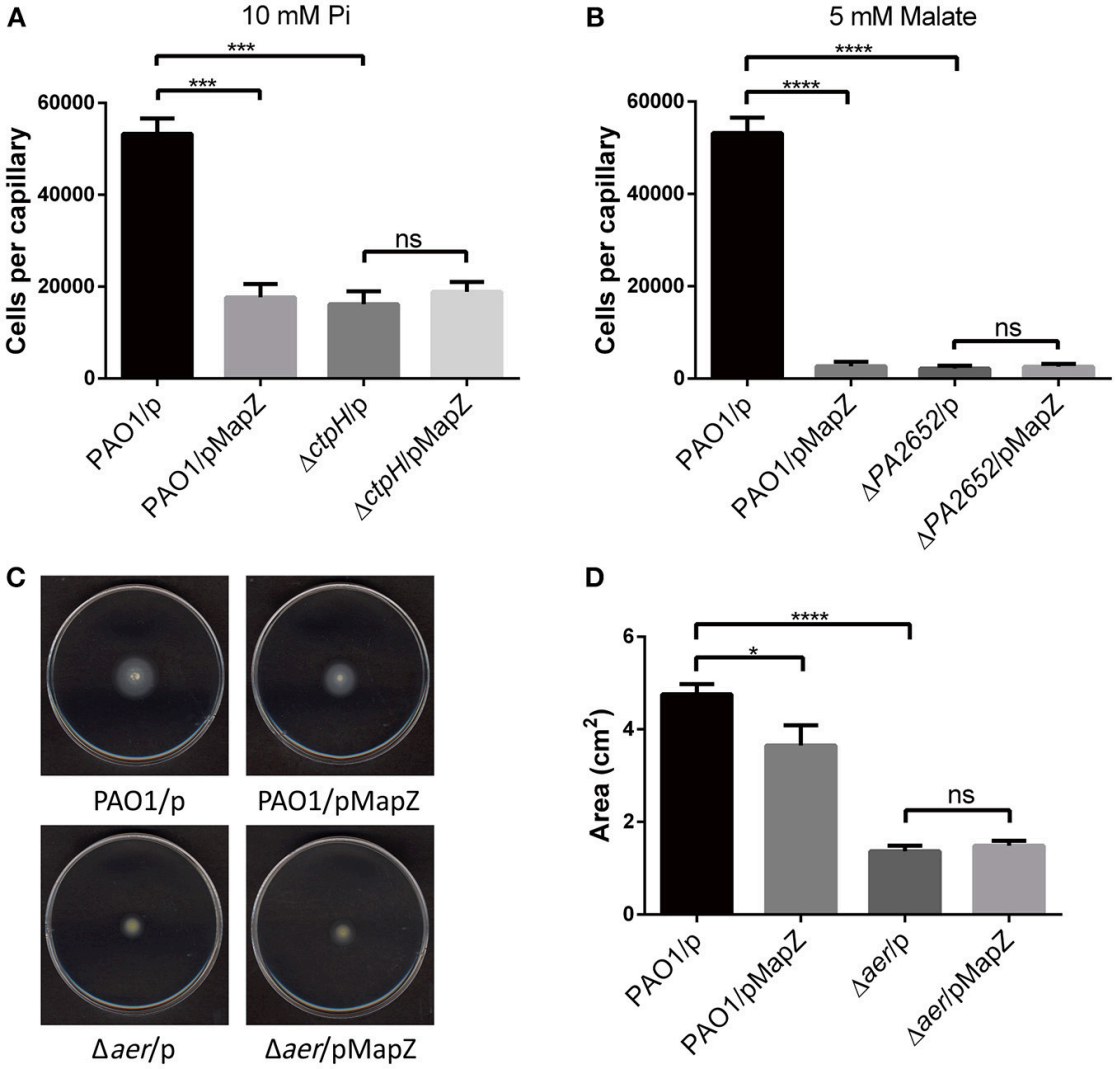
Chemotaxis and energy taxis assays demonstrating that MapZ affects the taxis toward inorganic phosphate, malate, and energy source. **(A)** Capillary chemotaxis assay of *P. aeruginosa* strains attracted toward 10 mM inorganic phosphate (Pi). The data were the means of three replicates and were normalized with the number of bacteria that swam into buffer-containing capillaries (Two-tailed *t*-test, ^***^P < 0.001). **(B)** Capillary chemotaxis assays of *P. aeruginosa* strains attracted toward 5 mM malate. The data were the means of three replicates and were normalized with the number of bacteria that swam into buffer-containing capillaries (Two-tailed *t*-test, ^****^P < 0.0001). **(C,D)** Energy taxis of *P. aeruginosa* strains on the minimal medium plates that contained 50 mM glucose as the sole carbon and energy source. The area of the swimming zone was quantified and compared in **(D)**. The data were the means of three replicates (Two-tailed *t*-test, ^*^P < 0.05).

We used the standard capillary chemotaxis assay to assess how overexpression of MapZ affects chemotaxis toward phosphate (Rico-Jimenez et al., 2016) and malate (Alvarez-Ortega and Harwood, 2007). We observed that the overexpression of MapZ in PAO1 significantly suppressed chemotaxis to inorganic phosphate (10 mM) and malate (5 mM), and that MapZ overexpression in the Δ*ctpH* and Δ*ctpM* mutant strains had no significant impact on the chemotactic response (Figures 3A,B). The observations indicate that MapZ impacts the chemotactic response to phosphate and malate via CtpH and CtpM, respectively. We also performed energy taxis assay to assess whether MapZ also affects energy taxis. Using the swimming plate assay to evaluate the response to nutrient (i.e., energy taxis; Nichols and Harwood, 2000), we observed that the swimming zone in minimal medium was significantly reduced when MapZ was overexpressed. In contrast, the swimming zone showed little difference when MapZ was overexpressed in the Δ*aer* strain (Figures 3C,D). The result confirms the role of Aer in energy taxis and suggest that MapZ is likely to play a role in energy taxis by controlling the methylation level of Aer.

### High Cellular Level of MapZ Impedes the Migration of *P. aeruginosa* to Wounded A549 Cells

**Figure 4:**
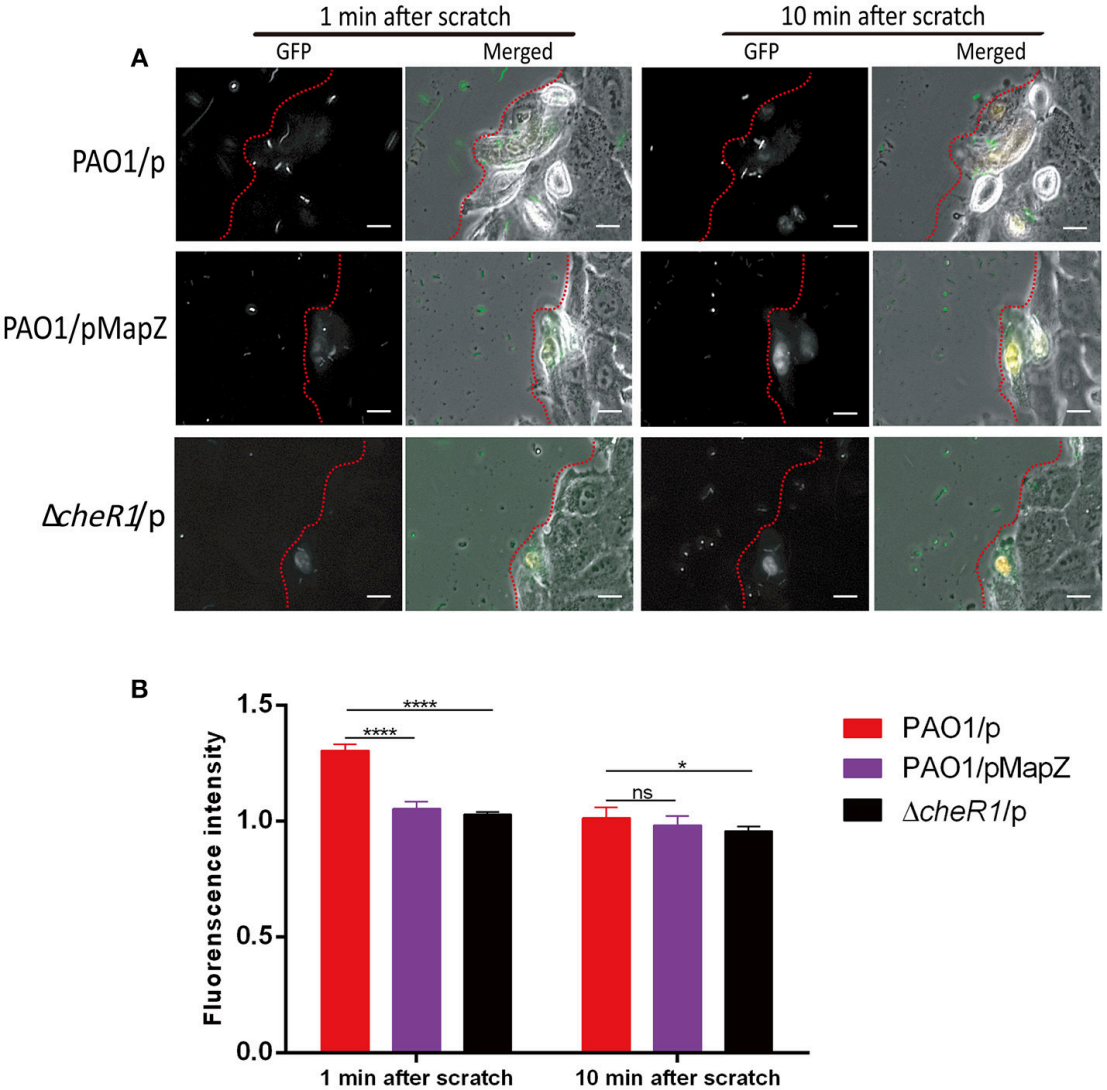
Chemotaxis-guided migration of *P. aeruginosa* strains toward scratch-wounded A549 human cells. **(A)** Representative microscopic images showing the accumulation of *P. aeruginosa* cells around wounded A549 human cells. The *P. aeruginosa* cells (in green) and wounded A549 cells (in white) are shown near the edge of the wound as indicated by the red dotted lines. The movies (Movies S1–S3) can be found in the supplementary material. Scale bar = 10 μm. **(B)** Quantitative comparison of the accumulation of *P. aeruginosa* cells around the injured A549 cells as indicated by fluorescence intensity. Three independent experiments were performed on each strain and at least 10 cells from each strain were used for quantitative analysis [Data are mean ± SD (*n* > 10)]. Two-tailed *t*-test, ^****^P < 0.0001, ^*^P < 0.05.

Flagellum-mediated chemotaxis plays an important role in pathogenesis because it is indispensable for the navigation and dissemination of *P. aeruginosa* cells in host (Matilla and Krell, 2018). Our data so far suggest that c-di-GMP MapZ-dependent pathway could influence the ability of *P. aeruginosa* to navigate and migrate in a host environment. We performed a scratch wound assay using human lung-derived A549 cells to test whether MapZ affects the ability of *P. aeruginosa* to migrate to wounded cells. In this assay, scratch-wounding a layer of A549 cells seeded on a cover-glass with a needle caused the immediate release of cellular contents, including nutrients such as free amino acids. It was expected that the *P. aeruginosa* cells with effective chemotactic pathways would respond to the chemicals released from the A549 cells by swimming rapidly toward the site of wounding of the dying cells. Indeed, we found that PAO1 cells migrated rapidly toward injured A549 cells within < 1 min to result in the accumulation of free-swimming bacteria near the site of wounding, as evidenced by increases in green fluorescence intensity near the wound (Figure 4A). By the end of the first few minutes, accumulation of PAO1 cells near the injured cells appeared to have reached an equilibrium due to the disappearance of chemical gradients. As negative controls, PAO1 cells did not migrate rapidly toward injured A549 cells within < 1 min in the presence of chemoattractants (inorganic phosphate, malate or amino acids), which can cause the lack of chemical gradient (Figure S1). We compared the behavior of the PAO1/pMapZ strain that has high cellular level of MapZ and the Δ*cheR1/p* strain to the PAO1/p strain. In contrast to PAO1, both the MapZ overexpression and Δ*cheR1/p* mutant strains showed weaker response in swarming to the wounded sites. There was significantly lower accumulation of bacterial cells near the sites of wounding for the mutant strain after the first minute post wounding (Figures 4A,B). Dispersion of the bacterial cells were observed after the few minutes due to the lack of chemical gradients, which resulted in a decrease in cell accumulation at the wound site at 10 min (Figures 4A,B). The observations suggest that the regulation of the chemosensory pathways by MapZ is important for the taxis of *P. aeruginosa* toward nutrients and other chemoattractants.

### MapZ-Mediated Regulation of Chemotaxis Contributes to Virulence in Acute Mouse Infection

Based on the results from the scratch-wound assay described above, we surmised that the MapZ could play an active role in pathogenesis. We first employed the nematode *C. elegans*, a simple and genetically tractable organism, to test how the overexpression of MapZ affect the ability of *P. aeruginosa* in infecting *C. elegans* under the fast-killing conditions (Mahajan-Miklos et al., 1999; Tan et al., 1999; Kirienko et al., 2014). By counting the number of live worms at different time points shown in Figure S2, we found that the death rate of *C. elegans* is about 10–30% lower for the MapZ overexpression mutant than that for PAO1 from 5 to 50 h after mixing *C. elegans* with PAO1 cells (Figure S2). It has been previously shown that virulence factors important for killing *C. elegans* are relevant for virulence in mammalian hosts (Mahajan-Miklos et al., 2000; Ewbank, 2002). Our results indicated that MapZ may also play an important role in the infection of mice and other mammalian hosts.

**Figure 5:**
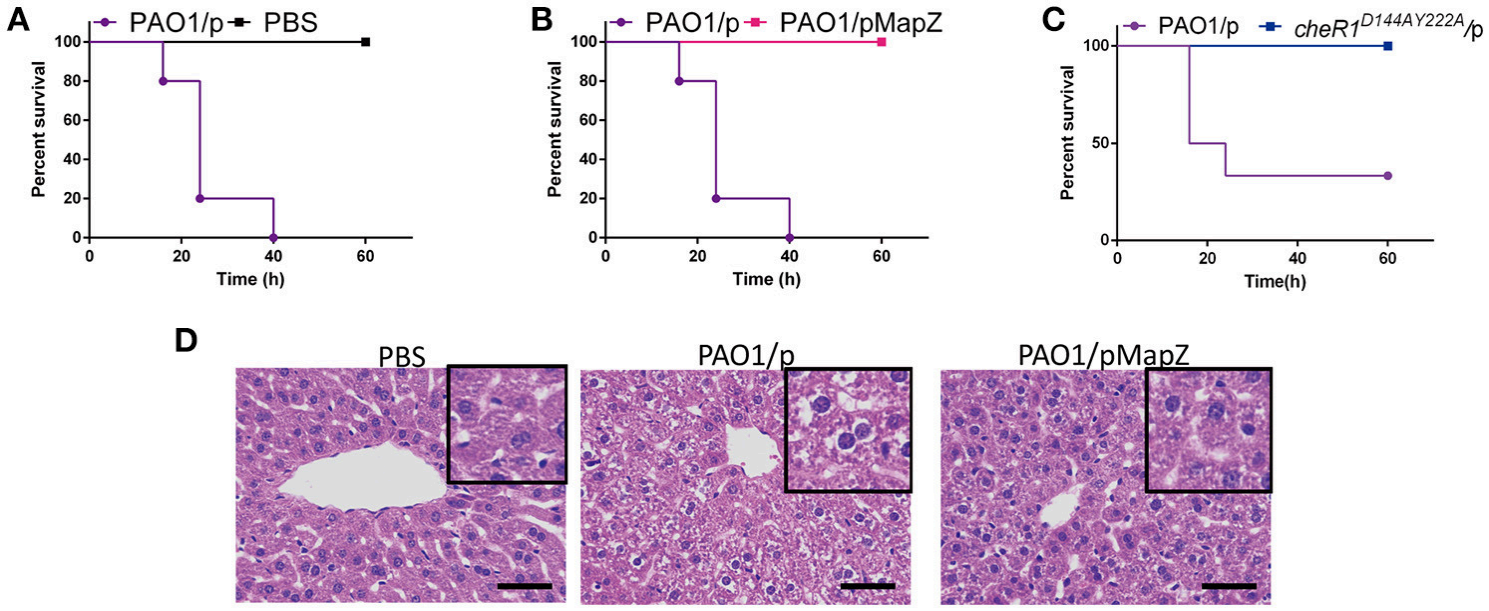
Overexpression of MapZ attenuated *P. aeruginosa* virulence. Survival rate of mice following intraperitoneal injection of 3.3 × 10^6^ CFU of PAO1/p and PBS **(A)**, 3.3 × 10^6^ CFU of PAO1/p and PAO1/pMapZ strains **(B)**, and 2.4 × 10^6^ CFU of PAO1/p and *cheR1*^*D*144*AY*222*A*^ strain **(C)**. Data are representative of three independent experiments with five mice used for each group. Histology of liver after intraperitoneal infections. Liver was harvested at the end of 24 h infection and processed for paraffin inclusion. Sections were stained with haematoxylin and eosin staining of liver tissue from the Babl/C mice. All cultures of the strains have the same OD_600_ at 0.1. **(D)** Representative photos for haematoxylin and eosin staining of bacteria-infected liver tissue from Babl/C mice after inoculation with PBS, PAO1/p, and PAO1/pMapZ for 24 h, respectively, PBS was used to be a negative control. Tissue infected with PBS can be seen a small amount of liver cells are mild edema around the central vein and edge with cell swelling and cytoplasm loose light dye. Tissue infected with PAO1/p is visible liver cells widely moderate edema with cell swelling and cytoplasm loose light dye, while tissue infected with mapZ_R13A/p, and PAO1/pMapZ only can be seen mild edema liver cells at part of the tissue and edge around the central vein with cell swelling and cytoplasm loose light dye. Data are representative of two independent experiments. Scale bar = 50 μm.

It was already shown recently that CheR1 and other chemotaxis genes are required for the fitness and virulence of *P. aeruginosa* in acute burn would infections (Turner et al., 2014). Considering the inhibition of CheR1 by MapZ and c-di-GMP, we reasoned that MapZ may play an important role in pathogenesis. We expected that PAO1/pMapZ strain that has high cellular level of MapZ will have the same virulence behavior as the *cheR1*^*D*144*AY*222*A*^ strain that harbors a chromosomal mutation (D144A-Y222A) to abolish the S-adenosylmethionine (SAM)-binding ability of CheR1 (Yan et al., 2018). To test this, we performed mouse infection experiment with *P. aeruginosa* strains injected intraperitoneally into the lower right quadrant of the abdomen of mice (Ozer et al., 2005). We found that all the mice infected with the PAO1 strain died by the end of 40 h (Figure 5A). Remarkably, none of the mice infected with the PAO1/pMapZ strain and *cheR1*^*D*144*AY*222*A*^ strain died by the end of 60 h (Figures 5B,C). These observations suggested that excess of MapZ attenuates the virulence of *P. aeruginosa* in mice. Furthermore, we found that intraperitoneally injection of PAO1, but not the overexpression strain PAO1/pMapZ caused histopathological changes and extensive necrosis of hepatocytes in the liver tissue (Figure 5D), which are likely to be caused by the immune response of the liver to the inflammatory microbial antigens or extracellular substrates produced by *P. aeruginosa*. Together, the observations indicate that a dysfunctional MapZ pathway attenuates the pathogenicity or virulence of *P. aeruginosa* and hinders the accumulation of *P. aeruginosa* in liver.

### Discussion

c-di-GMP has arisen in the last decade as a major regulator of pathogenicity and multicellular behavior of *P. aeruginosa* and other bacteria. Building on the recent findings that c-di-GMP can affect chemosensory pathways through the adaptor protein MapZ, this work demonstrates that the c-di-GMP-binding adaptor MapZ controls the methylation of multiple MCPs and is likely to exert a broad control on chemotaxis. MCPs control flagellar output in response to changing attractant or repellent concentrations and contribute to the pathogenicity of *P. aeruginosa* and other bacteria (Li et al., 2014; Choi et al., 2015; Sampedro et al., 2015; Nishiyama et al., 2016). Some of the MCPs that are methylated by CheR1 are already implicated in the bacterial pathogenesis of *P. aeruginosa* (Schwarzer et al., 2016); and an implication of this study is that the MapZ-associated pathway can influence bacterial pathogenesis via the chemosensory pathways. Considering that MapZ is one of the most conserved non-essential genes in the genus of *Pseudomonas* (Dötsch et al., 2010), the MapZ-mediated regulation of chemotaxis is most likely to be operative in other *Pseudomonas* species as well.

The finding that more than one third of the MCPs are controlled by MapZ suggests a previously underappreciated role of the MapZ-mediated regulatory system in *P. aeruginosa*. The three MCPs PctA, B and C play essential roles in the taxis toward the 20 commonly occurring L-amino acids, which are the major nutritional cues in the environment. The observation that CheR1 methylates PctA and PctB, the two MCPs responsible for the detection of the majority of L-amino acids (Taguchi et al., 1997), suggests that the taxis toward L-amino acids is likely to be suppressed by the MapZ-mediated mechanism under certain conditions. Apart from the MapZ-mediated regulation of chemotactic responses to amino acids, the two MCPs (CtpM and CtpH) responsible for detecting malate and inorganic phosphate are also methylated by CheR1. Malate is a simple organic acid and also serves as a nutritional cue; whereas the inorganic phosphate is an environmental signal that induces profound changes in gene expression and virulence expression in *P. aeruginosa* (Zaborin et al., 2009; Bains et al., 2012). It was proposed that the concentration of exogenous phosphate becomes limited at late stages of infection. *P. aeruginosa* uses phosphate deficiency as an environmental signal to trigger rhamnolipids production and shift from non-virulent to virulent phenotypes (Zaborin et al., 2012; Blus-Kadosh et al., 2013). *P. aeruginosa* and other Pseudomonas species have another signaling pathway (Pho signaling pathway) for detecting the availability of phosphate in the surroundings. Through the Pho two-component signaling pathway, *P. aeruginosa* responds to phosphate availability by adjusting cellular c-di-GMP concentration (Monds et al., 2010). Hence, the finding that MapZ controls the methylation and function of the phosphate-detecting CtpH suggests a two-tier mechanism whereby phosphate availability modulates chemotactic response directly through the CtpH chemosensory pathway and indirectly through the Pho/CtpH signaling pathways.

Aerotaxis is the movement of cells toward or away from oxygen, a response also termed energy taxis. Bacteria use aerotaxis to swim toward an optimal oxygen concentration for their metabolism. *P. aeruginosa* also has a strong energy taxis or aerotaxis response that is mediated mainly by the MCP Aer (Hong et al., 2004). Aer is also required for full tactic responses to some metabolizable compounds under both aerobic and anaerobic denitrifying conditions (Hong et al., 2004). The finding that a functional defect in MapZ affects the methylation of the aerotaxis chemoreceptors Aer indicates that MapZ-mediated mechanism is likely to govern the taxis toward O_2_-rich sources. In a recent study, it was found that the aerotaxis of the alpha-proteobacterium *Azospirillum brasilense* is regulated by intracellular c-di-GMP concentrations (O'Neal et al., 2017). However, unlike *P. aeruginosa* that relies on the discrete single PilZ domain protein MapZ, c-di-GMP mediates the changes in the aerotaxis response of *A. brasilense* by binding to the C-terminal PilZ domain of the chemotaxis receptors Tlp1. The occurrence of stand-alone and fused PilZ adaptor domains highlights the diverse mechanisms used for c-di-GMP to regulate chemosensory pathways.

Several studies already implicated the chemosensory pathways in the pathogenesis of *P. aeruginosa*. It was shown that the Che pathway is essential for the attraction of *P. aeruginosa* to scratch-wounded epithelial cells and subsequent immobilization of the bacterial cells at the wounded sites (Schwarzer et al., 2016). In particular, the three MCPs for amino acid detection (PctA/B/C) were found to play crucial roles in guiding *P. aeruginosa* to the wounded sites (Schwarzer et al., 2016). It was also demonstrated that the *P. aeruginosa* strain that lacks CheB2 exhibited attenuated virulence in nematodes (Garvis et al., 2009). The attenuated virulence of the Δ*cheB2* mutant was also demonstrated using a mouse lung infection, with the reduced virulence attributed to the failure of the mutant to induce strong inflammatory responses (Garvis et al., 2009). Our *C. elegans* and mouse infection studies strengthen the conclusion that the chemosensory pathways contribute to the pathogenicity of *P. aeruginosa* and are important for the pathogenesis process. The observation that a dysfunction of MapZ attenuated the virulence of *P. aeruginosa* indicates that the MapZ-mediated pathway is important for effective chemotaxis and pathogenesis. Given the role of c-di-GMP in regulating biofilm formation, we also tested how the overexpression of MapZ may affect *in vivo* biofilm formation using an established mouse infection model (Chua et al., 2016). However, the observed differences in biofilm accumulation and virulence between the WT and MapZ overexpression strains were not significant enough for us to draw any concrete conclusion (data not shown). Although whether the MapZ-mediated mechanism plays a role during *in vivo* biofilm formation and dispersal remains uncertain, the current results disclose the important role played by the MapZ-mediated mechanism in controlling chemosensory pathways and bacterial virulence.

## Materials and Methods

### Bacterial Strains and Plasmids

All bacterial strains and plasmids used in this study are listed in Table S1. Some of the Pseudomonas PAO1 mutant strains shown in Table S2 used in this study were obtained from the *P. aeruginosa* PAO1 transposon mutant library (Jacobs et al., 2003). The growth media used include Luria-Bertani broth, LB agar, 2 × YT broth, T_0_ broth, Minimal Salts agar, PGS agar and *Pseudomonas* isolation agar. For *P. aeruginosa*, all antibiotics were used at the following concentrations: carbenicillin at 300 μg/ml in all medium. For *E. coli*, all antibiotics were used at the following concentrations: ampicillin at 100 μg/ml in LB and kanamycin at 50 μg/ml in LB.

Gene-overexpression was accomplished by using the pUCP18 vector. All constructs were confirmed by DNA sequencing. Primers used for PCR and strain construction are listed in Table S2. Plasmids were introduced into *E. coli* and *P. aeruginosa* via electroporation and conjugation, respectively.

### Cloning and Expression of MCPs in *E. Coli*

The DNA fragments that encode 23 MCPs in Figure 1 were amplified using the primers listed in Table S2 with genomic DNA of *P. aeruginosa* PAO1 as template. Twenty one of the PCR products digested with the respective enzymes as shown in primers in Table S2 were cloned into pHSe5 with an IPTG induced promoter by T4 ligase methods except for *pctB* and *PA2654*, which were cloned into pHSe5 using the Gibson assembly method with kit from New England Biology Lab (NEB). All plasmids were transformed into *E. coli* HCB721 strain for overexpression of MCP proteins used for *in vitro* methylation assay.

### MCP Membrane Fraction Preparation

Membrane fractions containing MCP proteins were prepared as described (Schmidt et al., 2011; Xu et al., 2016). HCB721 is deficient in all *E. coli* MCPs and cytoplasmic chemotaxis proteins except for the CheZ phosphatase. Cells were grown at 37°C in 150 ml LB supplemented with 100 μg/ml ampicillin. MCP expression was induced with 1 mM IPTG at OD_600_ 0.5–0.7 for 3 h at 30°C, cells were harvested by centrifugation and re-suspended in the buffer containing 100 mM potassium acetate, 50 mM HEPES, pH 7.5, 5 mM magnesium acetate, 0.05% (v/v) β-mercaptoethanol, protease inhibitors (Complete mini, EDTA free, Roche), and Benzonase Nuclease (Novagen). Cells were lysed by sonication and cell debris was removed by centrifugation for 20 min at 7,000 × g, the supernatant was loaded on a sucrose step gradient (0.5, 1.5, and 2 M) and centrifuged at 100,000 × g for 1 h at 4°C. The second band was removed, diluted with water containing protease inhibitors (Complete Mini, with EDTA, Roche) and centrifuged for 1 h at 100,000 × g at 4°C. The pellets were resuspended in a small amount of storage buffer (50 mM NaH_2_PO4, 1 mM EDTA, 10% (v/v) glycerol). The membrane fraction samples were stored as single-use aliquots at 70°C.

### Preparation of Recombinant MapZ and CheR1 Proteins

Following the protocol described previously (Xu et al., 2016), the plasmids pET28b-MapZ and pET28b-CheR1 were transformed into the *E. coli* BL21(DE3) cell line. Fresh LB medium supplemented with kanamycin (50 μg/ml) was inoculated with the overnight culture. The temperature was reduced from 37 to 16°C when OD_600_ of the culture reached 0.6–0.8; and protein expression was induced by adding IPTG (1 mM). After 20 h, cells were harvested by centrifugation. Pellets were resuspended in PBS buffer (pH 7.4) that contained dithiothreitol and protease inhibitors. Cells were lysed by using the method of sonication. After centrifugation, the supernatant was filtered and incubated with Ni-NTA agarose beads (Qiagen). The beads were washed with PBS buffer (pH 7.4) that contained 10 mM imidazole. The recombinant protein was eluted with 400 mM imidazole-containing PBS buffer. The fractions from Ni-NTA column were pooled and concentrated before loaded onto an FPLC size-exclusion column (Sephedex) with PBS (pH 7.4). The fractions that contained the recombinant proteins were pooled and desalted using PD-10 Desalting Columns (GE Healthcare) and eluted using PBS buffer (pH 7.4). The proteins were concentrated, flash-frozen and stored at −80°C.

### *In vitro* Methylation Assays

Methylation assays were performed as previously described by Schmidt et al. (2011). Methylation assays of the MCPs by CheR1 were performed by measuring the transfer of radioactivity from the methyl donor [^3^H] Ado-Met SAM to MCP-containing membranes fraction. A reaction mixture (100 μl) containing 50 μl of MCP-containing membrane fraction and 0.1 μM purified 6 × His-CheR1 in reaction buffer [50 mM NaH_2_PO_4_ (pH 8.0); 300 mM NaCl)] was pre-incubated at 30°C for 10 min, and then 3 μl 0.625 μM [^3^H] Ado-Met (specific activity, 15 Ci/mmol; PerkinElmer) was added to the reaction mixture to initate methyl transfer reaction. After incubating at 30°C for 30 min the methylation reaction was stopped by adding 2 × SDS-PAGE loading dye. The reaction products were subjected to SDS-PAGE (12% polyacrylamide), and ^3^H-labeled MCP proteins were then visualized by autoradiography in each reaction. The effect of CheR1 (0.1 μM), MapZ (0.1 μM), c-di-GMP (10 μM) on methylation was examined by incubating the protein and c-di-GMP in the reaction mixture accordingly.

### Capillary-Based Chemotaxis Assays

Cells were grown in 2 × YT medium at 37°C with shaking overnight (Wu et al., 2000). T_0_ medium was inoculated with the overnight culture of *P. aeruginosa* PAO1 and incubated at 37°C for 4 h (Rico-Jimenez et al., 2016). The cells were washed twice with 10 mM HEPES (pH 7.0) and diluted to an OD600 of 0.04. Capillaries (0.3 mm) were sealed by flame at one end. With the sealed end remained warm, the open end was inserted into the chemoattractant to draw the solution. HEPES buffer containing capillaries were used as control. Diluted bacterial suspensions were placed onto Elisa plate, the capillaries were inserted into the wells and incubated for 30 min. After the incubation, the open end was rinsed with water, the sealed end was broken and the contents were transferred into 1 ml M9 medium supplemented with 15 mM succinate. After 10 × dilution, 20 μl of the cell suspension were spread onto agar plates containing M9 medium and succinate. Plates were incubated at 37°C for 24 h and then colonies were counted.

### Agar Plate-Based Energy Taxis Assays

Cultures (5–10 mL LB broth) were grown overnight with shaking. At OD_600_ 1.0, sterile toothpick was used to inoculate the minimal salts plate [3 g/L K_2_HPO_4_, 1.15g/L NaH_2_PO_4_, 1g/L NH_4_Cl, 0.15g/L MgSO_4_, 0.01g/L CaCl_2_, 0.0025g/L FeSO_4_, 0.3% agar, 50 mM glucose]. The plates were incubated for 24 h at 37°C before the diameters of the colonies were measured and pictures taken using an image scanner.

### Caenorhabditis Elegans Fast-Killing Assays

For growth rate characterization, *P. aeruginosa* strains cultivated in LB with carbenicillin (300 μg/ml) was monitored. Bacterial inocula at an initial turbidity of 0.2 (OD_600_) were added to the wells of a 100-well sterile plate (BIOSCREEN C) in 200 μl of LB with carbenicillin (300 μg/ml). The plates were measured at 37°C by BIOSCREEN C(Rossi-Rodrigues et al., 2009), and the OD_600_ was recorded every 2 h for up to 16 h.

For *Caenorhabditis elegans* fast-killing assay, we used PGS [1% Bacto-Peptone (BD Biosciences), 1% NaCl (Sigma), 1% glucose (Sigma), 0.15 M sorbitol, 1.7% Bacto-Agar (BD Biosciences)] medium as described previously (Tan et al., 1999). An overnight LB culture (5 μl) of the test bacterial strain was spread on a 3.5-cm diameter PGS agar plate, and incubated at 37°C for 24 h. The *E. coli* OP50 strain was used as the negative control. After 8–12 h at 20°C, each plate was seeded with 30 L4 stage worms (N2 Bristol). Plates were incubated at 20–25°C and scored for live worms every 4–6 h. A worm was considered dead when it no longer responded to touch on various part of the body. Time points were taken over a span of 50 h. The percentage of alive worms was calculated for each plate based on the counting of the dead and alive worms.

### Scratch Wound Assays

*P. aeruginosa* cells were grown to OD_600_ = 0.6 in LB broth at 37°C with agitation. The LB was removed and replaced with Hank's Balanced Salt Solution (HBSS) (ThermoFisher Scientific, USA) with tryptone (1% w/v). For imaging purpose, bacteria cells were stained with Vybrant™ DyeCycle™ Green Stain (ThermoFisher Scientific, USA) for 30 min at 37°C. Prior to imaging, the bacteria cells were washed with HBSS with tryptone to remove the excess cell stain. Propidium Iodide (1 μM) was added to identify damaged cells near the site of wounding. A549 cells grown in Dulbecco's Modified Eagle Medium (DMEM) (ThermoFisher Scientific, USA) were seeded to 100% confluency overnight and washed with HBSS. Bacteria cells suspension were added into 35 mm glass bottom (Ibidi, Germany) containing A549 cells, and equilibrated for 5 min before imaging. Phase contrast and fluorescence images were captured before wounding using the Zeiss Axiovert microscope (Carl Zeiss Microscopy, Jena, Germany) with a 20 × 1.4 NA objective. A universal 10 ul pipette tip was used to scrap the surface of the glass bottom dish to create a scratch wound. A549 cells were identified with Phase contrast images, green wild type PAO1 and mutant were imaged at EX:488 nm and PI-stained damaged cells at 561 nm. Time-lapse imaging was performed at every 30 s for 15 min. Fluorescence densitometric analyses were based on at least 10 cells per wild type and mutant bacteria cells for each condition. Axiovision 4.7 was used to quantify the fluorescence intensity around the wounded site. Data were normalized to pre-wounding condition into arbitrary values to illustrate relative changes in fluorescence intensity.

### Mouse Infection by Intraperitoneal Injection

Mouse infection by intraperitoneal injection assay was performed as previously described (Ozer et al., 2005). The mice used for the infection experiment were Male Babl/C mice (8–10 weeks old, weighing 18–21 g), which obtained from Guangdong animal experiment center. All mice in these experiments were allowed access to food and water *ad libitum*. Overnight cultures of *P. aeruginosa* were subcultured to OD_600_ = 0.2–0.4. Cultures were washed three times and diluted to OD_600_ = 0.2 in cold PBS. Bacterial samples were kept on ice until injection. Colony forming units (CFUs) were quantified by standard plate counting assay. Mice were injected intraperitoneally with 0.2 mL of the diluted *P. aeruginosa* suspension. Mice were carefully monitored and euthanized when end-point conditions were met (moribund, distressed, and unable to eat or drink).

Formaldehyde (4%)-fixed mouse livers were processed for histopathology as previously described (Leyva-Grado et al., 2017). Liver sections were stained with Hematoxylin—eosin (H&E). All slides were observed using NIKON Eclipse ci and NIKON digital sight DS-FI2. We examined two mice per bacterial group. The slides were evaluated by two experienced pathologists independently in a blinded fashion in order to confirm consistent adherence to the analytical criteria.

## Ethics Statement

This study was carried out in accordance with the recommendations of 3R and welfare principles of Experimental Animal Ethics Committee of South China Agricultural University. The protocol was approved by Experimental Animal Ethics Committee of South China Agricultural University.

## Author Contributions

SS, Z-XL, and LXu designed the study and wrote the manuscript with the assistance from other authors. SS, LXi, and RC cloned the MCPs and performed the methylation assays. SS, QL, and LXu performed the *C. elegans* fast-killing assays and intraperitoneal mouse infection assays. SS performed the chemotaxis and energy taxis assays. NK and HL performed the wound-scratch assays. JY, MS, and LY performed the implant mouse infection assays.

### Conflict of Interest Statement

The authors declare that the research was conducted in the absence of any commercial or financial relationships that could be construed as a potential conflict of interest.
