# Supplementary material for: The MapZ-Mediated Methylation of Chemoreceptors Contributes to Pathogenicity of Pseudomonas aeruginosa
Source: Front Microbiol. 2019 Feb 5;10:67. doi: 10.3389/fmicb.2019.00067 (PMC6370697; doi:10.3389/fmicb.2019.00067)
Supplement: Supplementary file 4 [file Data_Sheet_1.pdf]

Supplementary Materials

The MapZ-mediated Methylation of Chemoreceptors Contributes to Pathogenicity of *Pseudomonas aeruginosa*

Shuo Sheng[1,5], Lingyi Xin[2], Joey Kuok Hoong Yam[2,3,4], May Margarette Salido[2,3,4], Nicole Zi Jia Khong[2], Qiong Liu[1,5], Rachel Andrea Chea[2], Hoi Yeung Li[2], Liang Yang[2,3,4], Zhao-Xun Liang[2,4*], Linghui Xu[1, 6*]

1 Supplementary Movies, Tables and Figures.

1.1 Supplementary Movies

Supplementary Movie S1. The migration of PAO1 towards wounded A549 cells.

Supplementary Movie S2. The migration of PAO1/pMapZ towards wounded A549 cells.

Supplementary Movie S3. The migration of Δ*cheR*1/p towards wounded A549 cells.

1.2 Supplementary Tables

Supplementary Table S1

Supplementary Table S2

1.3 Supplementary Figures

Supplementary Figure S1

Supplementary Figure S2

Table S1. Bacterial strains and plasmids

**Strain or plasmid**	**Relevant genotype or description**	**Reference(s) or source**
	Escherichia coli	
E. coli BL21 (DE3)	Cells for high level expression of heterologous proteins in *E. coli*	Stratagene
E. coli HCB721	Cells for expression of *P. aeruginosa* chemoreceptors	(Wolfe et al., 1988)
E. coli DH5α	Cells for heat shock transformation	Transgen biotech
E. coli OP50	The food source used for *C. elegans* in fast-killing assay	Lab stock
	Pseudomonas aeruginosa	
PA01	Wild type	(Jacobs et al., 2003)
PA01/p	PAO1 strain containing pUCP18, Car^r	This study
PA01/pMapZ	PAO1 strain containing pUCP18-MapZ, Car^r	This study
mapZ_R13A strain containing pUCP18, Car^{r}Δ*cheR1*	PA3348 transposon mutant PW6640 from the Washington Genome Center PAO1 mutant library	(Jacobs et al., 2003)
Δ*cheR1/p*	Δ*cheR1* strain containing pUCP18, Car^r	This study
cheR1^{D144AY222A}	PAO1 strain containing D144A and Y222A mutation of PA3348 in genome	This study
cheR1^{D144AY222A}/p	*cheR1*^{D144AY222A} strain containing pUCP18, Car^r	This study
Δ*ctpH*	PA2561 transposon mutant PW5300 from the Washington Genome Center PAO1 mutant library	(Jacobs et al., 2003)
Δ*ctpH*/p	Δ*ctpH* strain containing pUCP18, Car^r	This study
Δ*ctpM*	PA2652 transposon mutant PW5440 from the Washington Genome Center PAO1 mutant library	(Jacobs et al., 2003)
Δ*ctpM*/p	Δ*ctpM* strain containing pUCP18, Car^r	This study
Δ*aer*	PA1561 transposon mutant PW3811 from the Washington Genome Center PAO1 mutant library	(Jacobs et al., 2003)
Δ*aer*/p	Δ*aer* strain containing pUCP18, Car^r	This study
	**Plasmids**	
pUCP18	*E. coli - P. aeruginosa* expression vector, Amp^r	Lab stock
pHSe5-PctA	PA4309 gene with stop codon cloned into pHSe5 vector, inducible with IPTG, Amp^r	(Schmidt et al., 2011)
pHSe5-PctB	PA4310 gene with stop codon cloned into pHSe5 vector, inducible with IPTG, Amp^r	This study
pHSe5-PctC	PA4307 gene with stop codon cloned into pHSe5 vector, inducible with IPTG, Amp^r	This study
pHSe5-CtpL	PA4844 gene with stop codon cloned into pHSe5 vector, inducible with IPTG, Amp^r	This study
pHSe5-Aer	PA1561 gene with stop codon cloned into pHSe5 vector, inducible with IPTG, Amp^r	This study
pHSe5-CtpH	PA2561 gene with stop codon cloned into pHSe5 vector, inducible with IPTG, Amp^r	This study
pHSe5-PA0411	PA0411 gene with stop codon cloned into pHSe5 vector, inducible with IPTG, Amp^r	This study
pHSe5-WspA	PA3708 gene with stop codon cloned into pHSe5 vector, inducible with IPTG, Amp^r	This study
pHSe5-CttP	PA0180 gene with stop codon cloned into pHSe5 vector, inducible with IPTG, Amp^r	This study
pHSe5-TlpQ	PA2654 gene with stop codon cloned into pHSe5 vector, inducible with IPTG, Amp^r	This study
pHSe5-CtpM	PA2652 gene with stop codon cloned into pHSe5 vector, inducible with IPTG, Amp^r	This study
pHSe5-PA4520	PA4520 gene with stop codon cloned into pHSe5 vector, inducible with IPTG, Amp^r	This study

pHSe5-PA2788	PA2788 gene with stop codon cloned into pHSe5 vector, inducible with IPTG, Amp^r	This study
pHSe5-PA2867	PA2867 gene with stop codon cloned into pHSe5 vector, inducible with IPTG, Amp^r	This study
pHSe5-PA2920	PA2920 gene with stop codon cloned into pHSe5 vector, inducible with IPTG, Amp^r	This study
pHSe5-PA1608	PA1608 gene with stop codon cloned into pHSe5 vector, inducible with IPTG, Amp^r	This study
pHSe5-PA4915	PA4915 gene with stop codon cloned into pHSe5 vector, inducible with IPTG, Amp^r	This study
pHSe5-PA4290	PA4290 gene with stop codon cloned into pHSe5 vector, inducible with IPTG, Amp^r	This study
pHSe5-PA1251	PA1251 gene with stop codon cloned into pHSe5 vector, inducible with IPTG, Amp^r	This study
pHSe5-McpK	PA5072 gene with stop codon cloned into pHSe5 vector, inducible with IPTG, Amp^r	This study
pHSe5-PA4633	PA4633 gene with stop codon cloned into pHSe5 vector, inducible with IPTG, Amp^r	This study
pHSe5-PA2573	PA2573 gene with stop codon cloned into pHSe5 vector, inducible with IPTG, Amp^r	This study
pHSe5-PA1646	PA1646 gene with stop codon cloned into pHSe5 vector, inducible with IPTG, Amp^r	This study

Table S2. Primers used in this study

Primers	Sequence (5'-3')	Purpose
pctC-F	CGCGGATCCATGCTTCGCTCGCTGTCGTTTG	For construction of pHSe5-PctC
pctC-R	CCCAAGCTTTCAGATCTTGAAGCTGTCCACC	For construction of pHSe5-PctC
pctB-F	CAATTTCACACAGGAAACAGATGATCAAAAGTCTCAAGTTC	For construction of pHSe5-PctB
pctB-R	TTGACAGCTTATCATCGATATCAGATCTTGAAGCTGTC	For construction of pHSe5-PctB
ctpL-F	CGCGGATCCATGCGCCTCAAGCAACTCACCA	For construction of pHSe5-PA4844
ctpL-R	CCCAAGCTTTCACAGGCGGAAGGCCTGCACC	For construction of pHSe5-PA4844
Aer-F	CGCGGATCCATGCGCAACAATCAGCCCATCA	For construction of pHSe5-PA1561
Aer-R	CCCAAGCTTTCAGCGGTTGAAGCGCTCGACC	For construction of pHSe5-PA1561
ctpH-F	CGCGGATCCATGCCCGCCTCGCCGGGCCACA	For construction of pHSe5-CTPH
ctpH-R	CCCAAGCTTTCAGGCCCGCAGGCGTTGCAGG	For construction of pHSe5-CTPH
PA0411-F	CGCGGATCCATGAAGAAAATCAACGCAGGCA	For construction of pHSe5-PA0411
PA0411-R	CCCAAGCTTTCAGGCCTGCTCCACGCCCTCC	For construction of pHSe5-PA0411
wspA-F	CGCGGATCCGTGAAGAACTGGACTGTTCGCC	For construction of pHSe5-PA3708
wspA-R	CCCAAGCTTTCAGACTTTGAAGCGGGATACG	For construction of pHSe5-PA3708
cttP-F	CGCGGATCCATGCGCGAGTCCCTCGGTGTTT	For construction of pHSe5-PA0180
cttP-R	CCCAAGCTTTCAGAACAATTCCACTTCGCCC	For construction of pHSe5-PA0180
tlpQ-F	CAATTTCACACAGGAAACAGATGTTCCTTCGCCGCCTG	For construction of pHSe5-PA2654
tlpQ-R	TTGACAGCTTATCATCGATATCAGGCCTTGAACTG	For construction of pHSe5-

	TTCC	PA2654
ctpM-F	CGCGGATCCATGATGCGTCTGACCCTGAAAT	For construction of pHSe5-CTPM
ctpM-R	CCCAAGCTTTCAGATGCGGAACTGACCGACC	For construction of pHSe5-CTPM
PA4520-F	CGCGGATCCGTGAAGACCGTACTCTATCCCG	For construction of pHSe5-PA4520
PA4520-R	CCCAAGCTTCTACACCCGGAACGCGCCGATC	For construction of pHSe5-PA4520
PA2788-F	CGCGGATCCATGAACGAAAGCGTCGCCAGGG	For construction of pHSe5-PA2788
PA2788-R	CCCAAGCTTTCAGGTACGGAAGCGGCCGAGC	For construction of pHSe5-PA2788
PA2867-F	CGCGGATCCATGGGCACCTGGATCAGCGACA	For construction of pHSe5-PA2867
PA2867-R	CCCAAGCTTTCAGAGGCGTAGCTGGCCGATG	For construction of pHSe5-PA2867
PA2920-F	CGCGGATCCATGCTGCAATGGTTCGCTAACC	For construction of pHSe5-PA2920
PA2920-R	CCCAAGCTTTCAGACGCGGAAACGGCCGACC	For construction of pHSe5-PA2920
PA1608-F	CGCGGATCCATGTCTTTGCGCAGTATGCCCA	For construction of pHSe5-PA1608
PA1608-R	CCCAAGCTTTCAGACGACGAAGCGGGTGACC	For construction of pHSe5-PA1608
PA4915-F	CGCGGATCCATGCTCACTGGCGTCACGGTTC	For construction of pHSe5-PA4915
PA4915-R	CCCAAGCTTCTAGACGGTGAAGCGCTGGATC	For construction of pHSe5-PA4915
PA4290-F	CGCGGATCCATGCAGCCCGCTCGTTCCCGCA	For construction of pHSe5-PA4290
PA4290-R	CCCAAGCTTCTAGCCGTTCAAGGCCAGGCTC	For construction of pHSe5-PA4290
PA1251-F	CGCGGATCCATGCTTCTTCGTCGTATCGCCA	For construction of pHSe5-PA1251
PA1251-R	CCCAAGCTTTCAGACCACGAAGCGACCGATC	For construction of pHSe5-PA1251
mcpK-F	CGCGGATCCATGTACGATTGGTGGGTTCTCC	For construction of pHSe5-PA5072
mcpK-R	CCCAAGCTTTTACAGGCGGAAGCGTCCCACC	For construction of pHSe5-PA5072
PA4633-F	CGCGGATCCATGAAGCTCAAGTCGATCCAGT	For construction of pHSe5-PA4633
PA4633-R	CCCAAGCTTCTAGACCCTGAACTGATTCACC	For construction of pHSe5-PA4633
PA2573-F	CGCGGATCCATGAACATTCGGCAGAGAATCC	For construction of pHSe5-PA2573
PA2573-R	CCCAAGCTTTCAGATCTTGAAGCGCTCCACC	For construction of pHSe5-PA2573
PA1646-F	CGCGGATCCATGCTGGGGTTGCTGCGCAGGC	For construction of pHSe5-PA1646
PA1646-R	CCCAAGCTTCTACAAGCGGAAATGCCTGACC	For construction of pHSe5-PA1646
pHSe5-F	CTGTTGACAATTAATCATCG	Detection primer of pHSe5
pHSe5-R	CTTTCGTCTTCAAGCAGATC	Detection primer of pHSe5

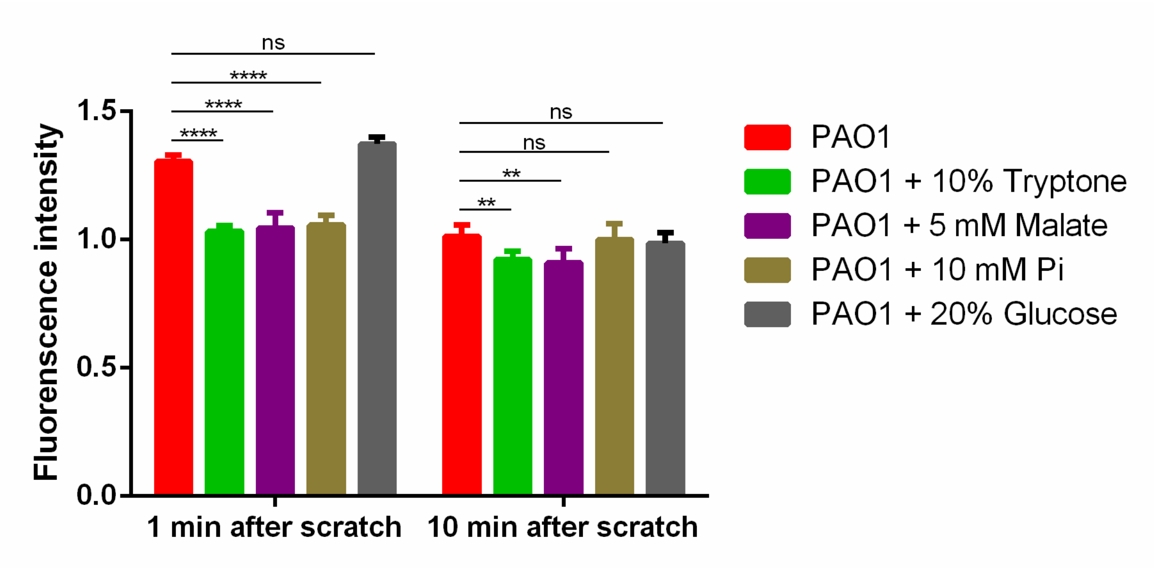

Figure S1. Chemotaxis-guided migration of *P. aeruginosa* strain PAO1 towards scratch-wounded A549 human cells in the presence of different chemoattractants. Quantitative comparison of the accumulation of *P. aeruginosa* cells around the injured A549 cells as indicated by fluorescence intensity. Three independent experiments were performed on each strain and at least 10 cells from each strain were used for quantitative analysis (Data are mean ± SD (n>10)).

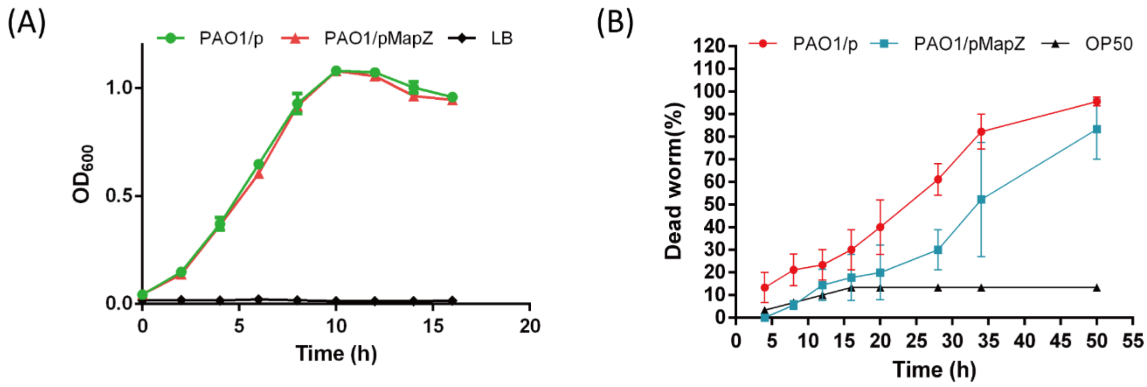

Figure S2. Overexpression of MapZ attenuated virulence in fast killing of the nematode *C. elegans*. (A) Growth curves of *P. aeruginosa* PAO1 and MapZ overexpression mutant in LB medium. The data are presented as the means ± standard errors from three independent experiments. (B) Overexpression of MapZ negatively impact virulence in fast-killing *C. elegans*. The data are presented as the means± standard errors from three independent experiments.
